# Numerical Analysis of Bead Magnetophoresis from Flowing Blood in a Continuous-Flow Microchannel: Implications to the Bead-Fluid Interactions

**DOI:** 10.1038/s41598-019-43827-x

**Published:** 2019-05-13

**Authors:** Jenifer Gómez-Pastora, Ioannis H. Karampelas, Eugenio Bringas, Edward P. Furlani, Inmaculada Ortiz

**Affiliations:** 1 Department of Chemical and Biomolecular Engineering, ETSIIT, University of Cantabria, Avda. Los Castros s/n, 39005 Santander, Spain; 2 Flow Science, Inc, Santa Fe, New Mexico 87505 USA; 3 Department of Chemical and Biological Engineering, University at Buffalo (SUNY), Buffalo, New York 14260 USA; 4 Department of Electrical Engineering, University at Buffalo (SUNY), Buffalo, New York 14260 USA

**Keywords:** Infection, Magnetic devices

## Abstract

In this work, we report a numerical flow-focused study of bead magnetophoresis inside a continuous-flow microchannel in order to provide a detailed analysis of bead motion and its effect on fluid flow. The numerical model involves a Lagrangian approach and predicts the bead separation from blood and their collection into a flowing buffer by the application of a magnetic field generated by a permanent magnet. The following scenarios are modelled: (i) one-way coupling wherein momentum is transferred from the fluid to beads, which are treated as point particles, (ii) two-way coupling wherein the beads are treated as point particles and momentum is transferred from the bead to the fluid and vice versa, and (iii) two-way coupling taking into account the effects of bead volume in fluid displacement. The results indicate that although there is little difference in the bead trajectories for the three scenarios, there is significant variation in the flow fields, especially when high magnetic forces are applied on the beads. Therefore, an accurate full flow-focused model that takes into account the effects of the bead motion and volume on the flow field should be solved when high magnetic forces are employed. Nonetheless, when the beads are subjected to medium or low magnetic forces, computationally inexpensive models can be safely employed to model magnetophoresis.

## Introduction

Applications of superparamagnetic nano- and micron-sized particles have proliferated in recent years, most notably in fields related to biomedical science and technology. This is due in part to rapid advances in particle synthesis and functionalization, as well as to the outstanding features of these particles such as high surface area to volume ratio and biocompatibility, among others. As a result, a number of different processes have been developed where these materials are being used as catalysts^1,2^, adsorbents^3–5^ and photocatalysts^6^ for water treatment, sensors for the detection and quantification of different components in fluid phases^7^, and magnetic recording and data storage devices^8^, to name but a few. However, as previously noted, the majority of applications can be found in the field of biomedicine where they are routinely employed as carriers for the capture or release of various biomolecules^9,10^.

**Figure 1:**
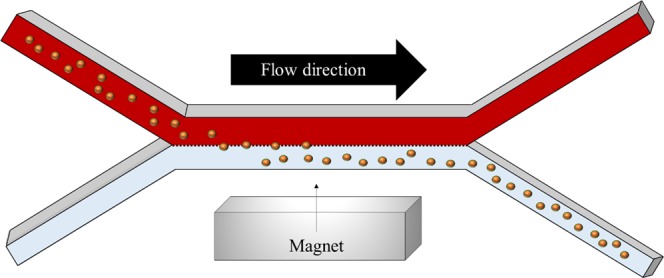
Sketch of the magnetophoresis process in the continuous-flow microdevice.

For most of these processes, the precise manipulation of magnetic particles, or micron-sized beads, using an applied magnetic field is of paramount importance. Microfluidic devices provide an especially good platform for such processes because of their many attractive features, e.g. laminar (non-turbulent) flow, which is readily controlled, small required samples, fast reaction rates, integration with multiple functionalities^11,12^. Consequently, many magnetophoretic microfluidic devices have been developed. These include magnetic separation devices working under different operation modes (i.e. batch or continuous), devices using different magnetic sources (i.e. permanent magnets, microelectromagnets or even superconducting magnets), and devices with different magnetic source combinations (i.e. active or passive configurations)^13–15^. Among the various types of microseparators, continuous-flow microsystems show different advantages in comparison to batch devices. In continuous-flow channels, the beads are deflected through multiple parallel streams by a magnetic gradient applied in a direction normal to the flow, as presented in Fig. 1, whereas in batch channels the beads are trapped at the high gradient regions, which are usually the channel walls. With continuous-flow channels, the flow restriction is minimized and thus, the overall efficiency and capacity of the separator is improved since the beads exit the device within a flowing phase. Furthermore, the use of multiple streams with different composition and parameters (chemical compounds, pH, biomolecules, etc.) in which the beads are deflected across, is an emerging and promising field because different steps such as capture, washing and analysis, can be integrated into the same device (i.e. lab-on-a-chip (LOC) and “micro total analysis systems” (µTAS))^16,17^.

However, continuous-flow magnetic separators must be properly designed to achieve complete bead deflection through the fluid solutions while eliminating or minimizing intermixing between the co-flowing streams as the beads cross the interface from one stream to its neighbor. This flow behavior could be difficult to achieve for some applications due to potential fluid perturbations caused by bead motion, especially under high magnetic forces^18^. Moreover, the colaminar streams should flow side-by-side and exit the channel independently, avoiding mixing between the fluids which would possibly cause loss or dissolution. Towards this goal, the flow characteristics of the streams need to be carefully examined. More specifically, the flow rates should be optimized in order to allow the bead deflection between phases, which could be achieved at low velocities, while minimizing any diffusion of the reagents between streams, which, on the contrary, might require relatively high flow rates.

While previous research has shown that micron-sized particles can be manipulated inside continuous-flow separators with simple permanent magnets, there has been relatively little work specifically focused on the effect of bead motion on fluid flow. This is due to the complexity of the mathematical description of this process^19^. Instead, only a few numerical studies of batch processes have been reported. For example, Khashan and Furlani^20^ reported the difference in particle separation effectiveness using one-way versus two-way coupling in a batch microfluidic system and found that, the results based on one-way coupling overpredict the magnetic force required for capturing the particles on the microchannel wall. Modak *et al*.^21^ also developed a method for estimating the capture of particles on straight and T-shaped batch microchannels, taking into account a two-way coupling by introducing the drag force exerted by the particle on the fluid to the momentum equation. Although these studies have demonstrated the relevance of the analysis of the fluid-particle interactions for accurate predictions of the magnetophoresis process, these works focus mostly on batch microchannels where the particles are trapped on the wall rather than deflected to another co-flowing phase. Hence, a flow-focused study of the magnetophoresis process for devices that involve the flow of multiple streams is currently lacking.

In our previous work^18^, we demonstrated that the complete bead deflection from different biological solutions can be achieved under different magnetic field conditions, but some of these conditions produce an unacceptable perturbation of the flow field. In that work, we employed a two-way coupling model that took into account the displacement of the fluid due to the bead motion. However, this model is computationally expensive, taking several weeks to run in a modern multicore workstation, and we did not quantify the difference in the results when simpler models are employed (one-way or two-way (treating beads as point particles)). Thus, an in-depth analysis of coupled bead-fluid interactions in continuous-flow microfluidic devices, reporting the effects of bead motion on the flow field as different effects are taken into account, has not yet been reported to the best of our knowledge.

Therefore, in this work we provide a detailed analysis of bead motion and its effect on fluid flow inside a continuous-flow magnetophoretic device where the beads are magnetically separated from a flowing biological fluid (blood) and recovered into an aqueous buffer solution, as shown in Fig. 1. Our numerical model describes the particle separation due to the presence of a magnetic field provided by a permanent magnet. Three different fluid perturbation scenarios are compared and discussed: (i) one-way coupling between the beads and the fluid wherein the fluid flow affects bead motion but the bead motion does not perturb the fluid flow, (ii) two-way coupling wherein there is a two-way momentum transfer between the beads and the fluid, i.e. the bead motion perturbs the fluid flow, and (iii) two-way coupling including fluid displacement dependent on the bead volume. Our analysis shows that, under certain circumstances, the magnetophoretic motion of the beads changes the flow patterns, and therefore, the effect of the particle motion on the fluid flow should be considered for certain magnetic conditions. Most importantly, we provide useful guidelines about the model to be chosen depending on the magnetic conditions inside the channel, in order to optimize the simulation runtimes without sacrificing results accuracy.

## Theory

### Approach

The computational model used in this work involves an Eulerian-Lagrangian approach to predict the magnetophoretic bead transport. For tracking the particle trajectories, we employ a Lagrangian approach whereas a Eulerian framework is employed to solve the fluid flow field. Moreover, the numerical model not only includes the major forces driving the separation, which are the magnetic and the fluidic forces, but also the effect of the particle motion on the fluid flow through three different scenarios. The bead-fluid coupling and transport is predicted using numerical computational fluid dynamic (CFD)-based analysis, whereas the magnetic force exerted on the particles is solved analytically. According to the aforementioned approaches, beads are considered discrete elements and their trajectories are estimated as follows:

1 $${{\rm{m}}}_{{\rm{p}}}\frac{{\rm{d}}{{\bf{v}}}_{{\bf{p}}}}{{\rm{dt}}}={\sum }^{}{{\bf{F}}}_{{\rm{ext}}}$$

where m_p_ and d**v**_**p**_/dt are the bead mass and acceleration, and **F**_ext_ is the resultant force vector exerted on a bead and can be written as:

2 $${\sum }^{}{{\bf{F}}}_{{\rm{ext}}}={{\rm{m}}}_{{\rm{p}}}{\bf{g}}+{{\bf{F}}}_{{\rm{hd}}}+{{\bf{F}}}_{{\rm{m}}}$$

where **g** represents gravity, **F**_hd_ is the hydrodynamic force acting on the beads and **F**_m_ is the magnetic force on the beads. In this work, the effects of gravity are small compared to the other forces and therefore neglected. In the following sections, the dominant force contributions are discussed.

### Magnetic force

The magnetic force **F**_m_ acting on a bead is predicted with the “effective” dipole moment method as discussed in our previous work^18^. According to this method, the magnetic material can be replaced by a point dipole with an equivalent moment **m**_p,eff_^22^. Therefore, the force acting on the material can be expressed as:

3 $${{\bf{F}}}_{{\rm{m}}}={{\rm{\mu }}}_{0}({{\bf{m}}}_{{\rm{p}},{\rm{eff}}}\cdot \nabla ){{\bf{H}}}_{{\rm{a}}}$$

where µ_0_ is the permeability of the free space (4π·10^−7^ H·m^−1^) and **H**_a_ is the applied magnetic field at the bead center. In this work, **m**_p,eff_ is calculated with a magnetization model that includes self-demagnetization and magnetic saturation of the beads^23^:

4 $${{\bf{m}}}_{{\rm{p}},{\rm{eff}}}={{\rm{V}}}_{{\rm{p}}}{{\rm{f}}({\rm{H}}}_{{\rm{a}}}){{\bf{H}}}_{{\rm{a}}}$$

where the function f(H_a_) is computed as:

5 $${{\rm{f}}({\rm{H}}}_{{\rm{a}}})=\{\begin{array}{cc}\frac{3({\chi }_{p}-{\chi }_{f})}{({\chi }_{p}-{\chi }_{f})+3} & \,|{{\rm{H}}}_{{\rm{a}}}| < (\frac{({\chi }_{p}-{\chi }_{f})+3}{3({x}_{p}-{x}_{f})}){{\rm{M}}}_{{\rm{s}},{\rm{p}}}\\ \frac{{{\rm{M}}}_{{\rm{s}},{\rm{p}}}}{|{{\rm{H}}}_{{\rm{a}}}|} & |{{\rm{H}}}_{{\rm{a}}}|\ge (\frac{({\chi }_{p}-{\chi }_{f})+3}{3({\chi }_{p}-{\chi }_{f})}){{\rm{M}}}_{{\rm{s}},{\rm{p}}}\end{array}$$

where V_p_ is equal to the volume of the beads, which are assumed spherical in this work, χ_p_ and χ_f_ represent the magnetic susceptibilities of the bead and the fluid, respectively, and M_s,p_ is the particle saturation magnetization.

Therefore, and after substituting the previous equations into Eq. (1), the force acting on the beads can be written as follows:

6 $${{\bf{F}}}_{{\rm{m}}}={{\rm{\mu }}}_{0}{{\rm{V}}}_{{\rm{p}}}{{\rm{f}}({\rm{H}}}_{{\rm{a}}})({{\bf{H}}}_{{\rm{a}}}\cdot \nabla ){{\bf{H}}}_{{\rm{a}}}$$

As it appears in Eqs (5) and (6), **F**_**m**_ depends on the properties of both the beads and the fluid phases. Regarding the bead properties, they are micron-sized with a diameter of 5 μm, a density of 2000 kg·m^−3^ and a saturation magnetization of 10^5^ A·m^−1^. Beads with such properties are commercially available and employed in relevant biological and biomedical studies^24,25^. Regarding the magnetic properties of the fluid phases, it should be known that blood cells (oxygen-depleted red blood cells) are considered magnetic as they can be magnetically separated from aqueous phases under strong magnetic field gradients^26,27^. However, the susceptibility of these cells in comparison to commercial magnetic beads is much lower (around −5 × 10^−6^ in SI)^26^, and thus, the possible magnetic force exerted on them would be several orders of magnitude lower than the force exerted on the beads. Therefore, and in order to simplify the analysis, the surrounding fluids are considered non-magnetic, and the following magnetization model was adopted^28,29^:

7 $${{\rm{f}}({\rm{H}}}_{{\rm{a}}})=\{\begin{array}{cc}3, & |{{\rm{H}}}_{{\rm{a}}}| < \frac{{{\rm{M}}}_{{\rm{s}},{\rm{p}}}}{3}\\ \frac{{{\rm{M}}}_{{\rm{s}},{\rm{p}}}}{|{{\rm{H}}}_{{\rm{a}}}|}, & |{{\rm{H}}}_{{\rm{a}}}|\ge \frac{{{\rm{M}}}_{{\rm{s}},{\rm{p}}}}{3}\end{array}$$

Finally, the magnetic field generated by the permanent magnet is required for calculating **F**_**m**_. The 3D magnetic field and gradient is analytically calculated by the model developed by Furlani^30^. A rare-earth magnet, with dimensions of 1 × 1 × 1 mm^3^, was selected for this study. Such magnets are also commercially available. The magnet was placed at a varying distance from the bottom channel, with its center aligned with the channel central depth.

### Fluidic force

The hydrodynamic force **F**_hd_ on the beads is predicted numerically using the following expression:

8 $${{\bf{F}}}_{{\rm{hd}}}=-\,{{\rm{V}}}_{{\rm{p}}}\nabla {\rm{P}}+{{\rm{M}}}_{{\rm{added}}}(\frac{{\rm{D}}{\bf{v}}}{{\rm{Dt}}}-\frac{{\rm{d}}{{\bf{v}}}_{{\rm{p}}}}{{\rm{dt}}})+{{\bf{F}}}_{{\rm{drag}}}$$

where P is the pressure, M_added_ is the added mass, **v** is the fluid velocity and **F**_drag_ is the drag force acting on the beads. The first term in Eq. (8) represents the pressure force acting on the bead due to a pressure differential. The added mass term can be described as an additional resistance for an accelerating or decelerating body. We note that the exact formulation for the added mass includes the total or full derivative following the fluid velocity (D**v**/Dt)^31^, as presented in Eq. (8). Nonetheless, the mathematical procedure for solving the full derivative of the fluid velocity is complex; thus, for simplicity, we resolved the partial derivative of the fluid velocity in the added mass term (∂**v**/∂t).

The amount of added mass can be calculated using the following equation:

9 $${{\rm{M}}}_{{\rm{added}}}={{\rm{C}}}_{{\rm{M}}}{{\rm{\rho }}{\rm{V}}}_{{\rm{p}}}$$

where the coefficient C_M_ is theoretically predicted to be equal to 0.5 and ρ is the fluid density^32^.

Finally, the drag force **F**_drag_ can be obtained using a modified form of Stokes’ approximation for the drag on a sphere:

10 $${{\bf{F}}}_{{\rm{drag}}}=\frac{1}{2}{\rm{\rho }}({\bf{v}}-{{\bf{v}}}_{{\rm{p}}})|{\bf{v}}-{{\bf{v}}}_{{\rm{p}}}|{{\rm{A}}}_{{\rm{p}}}{{\rm{C}}}_{{\rm{d}}}$$

where A_P_ is the bead cross sectional area, which can be written as A_P_ = πr_p_^2^. C_D_ is the drag coefficient for steady-state flow around a sphere and can be calculated from:

11 $${{\rm{C}}}_{{\rm{D}}}=\frac{24}{{{\rm{Re}}}_{{\rm{p}}}}+\frac{6}{1+\sqrt{{{\rm{Re}}}_{{\rm{p}}}}}+0.4$$

where Re_p_ is the particle Reynolds number for which the expression is valid^33^. More specifically, we have:

12 $${{\rm{Re}}}_{{\rm{p}}}=\frac{2{{\rm{\rho }}r}_{{\rm{p}}}|{\bf{v}}-{{\bf{v}}}_{{\rm{p}}}|}{{\rm{\eta }}} < 2\cdot {10}^{5}$$

where η is the fluid dynamic viscosity.

It should be noted that in Eq. (8) we did not account for the external force exerted by the shear stress on the particles (for Scenarios 1 and 2). This effect would cause particle rotation due to the difference in shear between neighboring mesh cells^34^. Furthermore, although the difference in pressure was taken into account to predict pressure forces, this effect could be considered negligible for the magnetophoresis process as it does not considerably modify the magnetophoretic velocity component in the direction normal to the flow.

To evaluate the previous equations, we need expressions for the fluid properties (density and viscosity) and their velocity distributions. One of the fluids in our analysis is blood with rheological properties that depend on many factors. These include the flow velocity, red blood cell concentration, channel diameter (Fahraeus-Lindqvist effect), size of the cells and their aggregation and deformation, etc., as previous studies have already reported^35^. Blood was modeled in this study as a Newtonian fluid with an average viscosity value equal to 3.5 cP. The viscosity was calculated by an analytical empirically based expression that takes into account the plasma viscosity, the width of the channel and the blood cell volume concentration^18^. It should be noted that it has been demonstrated in previous works^36^ that blood follows a Newtonian rheology when the shear rate exceeds about 100 s^−1^, and we have also experimentally validated this assumption^37^. Regarding the aqueous buffer solution, it was model as water with a constant viscosity equal to 1 cP. Fluid velocity and bead-fluid interactions are discussed in the following subsection.

### Fluid velocity field and bead-fluid interactions

The fluid velocity field was predicted using the Navier Stokes and continuity equations, to which certain modifications were applied in order to precisely describe the bead-fluid interactions. Particularly, three scenarios were modeled and compared. In the scenario 1, we did not take into account the effects of the bead motion or bead volume in the fluid flow field, but only the effect of the fluid motion on the magnetophoresis process (one-way momentum transfer from the fluid to the beads). This assumption implies that the particles are dragged by the fluid, but they do not affect the stream lines, which is the scenario usually employed in most numerical studies of magnetophoresis^38^. Thus, the fluid velocity field is estimated by Navier-Stokes and continuity equations (as presented in Eqs (13) and (14)), and no momentum transfer from the beads to the fluid is taken into account. This one-way coupling is solved here as a base case for comparison with the two-way coupling scenarios. The equations governing the one-way coupled incompressible flow are as follows:

13 $$\frac{{\rm{d}}({\rm{\rho }}{\bf{v}})}{{\rm{dt}}}=-\,\nabla {\rm{P}}+{\rm{\rho }}{\bf{g}}+\mathrm{div}({\boldsymbol{\tau }})$$

14 $$\nabla \cdot ({\bf{v}})=0$$

where the div(**τ**) term represents the contribution of shear stress on the fluid velocity. The flow equations are numerically solved by using the Volume of Fluid (VOF) method as implemented in the commercial **FLOW-3D** software. With this method, an additional equation is solved in order to calculate the volumetric fraction F of each fluid in every computational mesh cell, as follows:

15 $$\frac{\partial {\rm{F}}}{\partial {\rm{t}}}+\frac{1}{{{\rm{V}}}_{{\rm{f}}}}[\nabla ({\rm{F}}{\bf{A}}{\bf{v}})]=0$$

where V_f_ and **A** represent the fractional volume and fractional area open to flow for each mesh cell. Thus, the VOF function, F, represents the volumetric fraction of the incompressible phase 1 (blood), corresponding the complementary region with fraction 1-F, the volumetric fraction of phase 2 (buffer). It should be noted that the fluid properties (either density or viscosity) depend on the value of the VOF function. More specifically, the flow field equations within the VOF method are solved by using a volume-fraction-weighted density (ρ) and viscosity (μ) through the function F.

In the second scenario, we account for a two-way coupling, i.e. two-way momentum transfer between the moving particles and the fluid. However, the beads are still considered as point particles, i.e. we do not take into account the effects of the bead volume. Two-way coupling is taken into account by including the particle accelerations into the momentum equation, as follows:

16 $$\frac{{\rm{d}}({\rm{\rho }}{\bf{v}})}{{\rm{dt}}}=-\,\nabla {\rm{P}}+{\rm{\rho }}{\bf{g}}+\mathrm{div}({\boldsymbol{\tau }})+\frac{1}{{\rm{V}}}{{\bf{F}}}_{{\rm{P}}}$$

The last term in Eq. (16) represents particle induced fluid accelerations, where V is the volume of fluid in the cell and **F**_P_ can be written as:

17 $${{\bf{F}}}_{{\rm{P}}}=-\,{\sum }^{}[{{\bf{F}}}_{{\rm{drag}}}+{{\rm{M}}}_{{\rm{added}}}(\frac{{\rm{d}}{\bf{v}}}{{\rm{dt}}}-\frac{{\rm{d}}{{\bf{v}}}_{{\rm{p}}}}{{\rm{dt}}})]$$

Therefore, for a given control volume, Eq. (17) describes the contribution of the particles to the flow which is the summation of the drag and added mass terms for each bead present in the computational cell, i.e. the contribution of all beads present in the cell are cumulatively added to the momentum through **F**_p_ in Eq. (16).

For the third case, we take into account two-way coupling and the effects of the liquid being displaced by the bead volume. Thus, the continuity equation is altered to account for solid objects in the computational cells according to the FAVOR^TM^ method as follows^39^:

18 $$\frac{\partial }{\partial {\rm{t}}}({{\rm{\rho }}V}_{{\rm{f}}})+\nabla ({\rm{\rho }}{\bf{v}}{\bf{A}})={{\rm{S}}}_{{\rm{m}}}$$

where S_m_ is a physical mass source term of fluid which may be present in the cell. Thus, a solid object located inside a computational cell is taken into account by using a single volume fraction (V_f_) that describes how much of the cell volume is occupied by the solid and by 3 area fractions stored at the cell faces (**A**) that describe where the object is located. This method has advantages over the moving and deforming mesh methods conventionally applied in CFD for describing moving objects^40^. This is because it treats complex moving object geometries very efficiently and conveniently (not requiring automatic mesh regeneration techniques) and there is no restriction on closeness between objects. Nonetheless, it unfortunately does not allow for multiple solid surfaces inside the cell, i.e. cases where multiple particles are occupying the same cell cannot be accurately modelled; also, accuracy is affected for cases where the solid object contains peaks or corners since such geometrical features will be replaced by a single surface.

In contrast to stationary geometry problems, to account for moving objects the volume and area fractions vary with time and their effects on fluid flow must be considered. Therefore, the previous equation can be rewritten as follows:

19 $$\frac{{{\rm{V}}}_{{\rm{f}}}}{{\rm{\rho }}}\frac{\partial {\rm{\rho }}}{\partial {\rm{t}}}+\frac{1}{{\rm{\rho }}}\nabla ({\rm{\rho }}{\bf{v}}{\bf{A}})=-\,\frac{\partial {{\rm{V}}}_{{\rm{f}}}}{\partial {\rm{t}}}+\frac{{{\rm{S}}}_{{\rm{m}}}}{{\rm{\rho }}}$$

which for incompressible flow becomes:

20 $$\nabla ({\bf{v}}{\bf{A}})=-\,\frac{\partial {{\rm{V}}}_{{\rm{f}}}}{\partial {\rm{t}}}+\frac{{{\rm{S}}}_{{\rm{m}}}}{{\rm{\rho }}}$$

The term $-\frac{\partial {{\rm{V}}}_{{\rm{f}}}}{\partial {\rm{t}}}$ is equivalent to an additional source term. With the VOF method, this term appears only in mesh cells around the moving object boundary and, for a solid particle that is much bigger than a computational cell, it is calculated as follows:

21 $$\frac{\partial {{\rm{V}}}_{{\rm{f}}}}{\partial {\rm{t}}}=-\,\frac{{{\rm{S}}}_{{\rm{p}}}}{{{\rm{V}}}_{{\rm{cell}}}}{{\bf{v}}}_{{\rm{p}}}\cdot {\bf{n}}$$

where S_p_ is the surface area of the bead inside the computational cell (not to be confused with the area fraction **A**), and V_cell_ and **n** are the volume of the mesh cell and the unit normal vector in the mesh cell, respectively. The normal vector is added to the equation to describe cases where the surface of the bead does not follow the direction of its velocity, e.g. when the surface is tangential to the direction of the velocity of the object.

For the third case scenario, and only for this case where the beads are considered to occupy volume, the beads were allowed to move with six degrees of freedom (DOF), i.e. 3 rotations and 3 translations. In this transient analysis, the solver calculates all fluid forces and torques as well as the forces from the magnetic field for each time-step. For the moving beads, the equations of motion as well as the boundary conditions and supplemental variables that define their coupled motion (e.g. velocities at the boundaries, wetted areas, displaced fluid volume, etc.) are also calculated. Eqs (1) and (22) provide a general mathematical description for the motion of a rigid body in 6-DOF:

22 $${{\bf{T}}}_{{\rm{G}}}=|{{\rm{J}}}_{{\rm{m}}}|\cdot \frac{{\rm{d}}{\boldsymbol{\omega }}}{{\rm{dt}}}+{\boldsymbol{\omega }}\times (|{{\rm{J}}}_{{\rm{m}}}|\cdot {\boldsymbol{\omega }})$$

where **T**_G_ is the torque around its center of mass G, |J_m_| is the moment of inertia tensor in the body system and **ω** is the angular velocity of the rigid body. For the purpose of our analysis, the initial condition ω(t = 0) was set to zero, i.e. the beads are not rotating at the beginning of the simulation, but they are allowed to rotate depending on the flow conditions. It should be noted that, in general, the total torque can be divided into several net components (hydraulic, gravitational, magnetic, etc.). However, we did not include the effect of the magnetic field on the total torque in order to simplify the problem. This is because it would increase the computational cost of the simulations and the particle rotation information might not be meaningful as the translational movement (especially in z direction) is more important for the practical application of the device. Furthermore, the gravitational torque vanishes for beads with 6-DOF motion. Therefore, the only contribution on the torque in this work is due to the hydraulic component (**T**_G_ = **T**_hd_). This can be thought as rotation of the beads inside the device due to tangential shear forces acting on their surface. Since in this scenario the beads are modelled as rigid solid bodies with volume and mass, and since their radii cross multiple gridlines, the velocities across the two opposite surfaces of the spherical bead will differ, because of the parabolic flow profile, with the exception of the bead center being located at the location of maximum velocity i.e. the tip of the parabolic flow profile. This asymmetry would, in turn, cause an imbalance of shear forces applied on each surface which, consequently, would cause the spherical bead to roll towards the tip of the parabolic flow profile. This phenomenon although modelled is not of primary concern since we consider its effect negligible compared to the overall translational magnetic forces. Finally, it should be noted that for scenarios 1 and 2 the beads are treated as points and the torque is not calculated.

### Methodology

**Figure 2:**
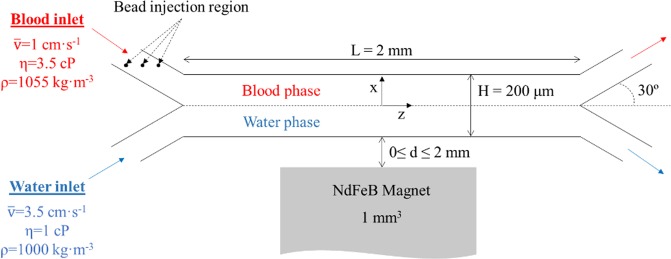
Schematic view of the microdevice showing the working conditions set in the simulations.

The microfluidic device modeled in this study comprises of a Y-Y shaped channel with a total height of 200 µm and length of 2 mm, as depicted in Fig. 2. Although the force balance for each bead is solved within a 3D analysis (the magnetic field distribution and magnetic force is calculated in x, y, and z directions), the governing equations for the flow along the width of the channel (y axis) are not considered for scenarios 1 and 2. For scenario 3, a full 3D model is developed and the extent of the computational domain in the y direction was set to a value equal to 3 times the bead diameter, in order to solve the particle movement in this direction. For this case, periodic boundary conditions are applied at both limits of the y direction. A uniform grid is employed for all the simulations. For scenarios 1 and 2 the mesh is composed by approximately 75,000 cells (2D simulations), whereas this number is increased to a range of 700,000–1,200,000 cells for scenario 3. The increased mesh size is necessary for accurate predictions of the bead volume within the fluid domain. It should be noted that, in a real application, the device performance would be controlled under a microscope. Under these circumstances, both fluids will flow in parallel at the same height with gravity acting on y direction. Therefore, it is assumed that blood and water flow at the same level and the effects of gravity are neglected since it does not act in the x-z plane shown in Fig. 2.

In order to study the magnetophoresis process under different magnetic field conditions, the distance between the top of the magnet and the wall of the channel was varied between 0 and 2 mm. In our previous work^18^, we demonstrated that in order to achieve complete separation between blood/buffer at the outlets, a ratio between the velocities should be kept at 3.5. Thus, for all the simulations the velocities of blood and water were kept at 1 and 3.5 cm·s^−1^, respectively. These velocity values were also used as initial conditions. No slip condition (zero velocity) is applied along the microchannel walls (due to the laminar regime) and at the outlet, the outflow boundary condition was employed (generally applied for confined incompressible fluid flows). It should be noted that, under this velocity conditions, the residence time of both phases inside the separator is less than 1 second, which avoids interdiffusion of species between the streams at the time scales of interest^41^. Multiple cases were investigated by keeping the average hydrodynamic forces at a constant value and by varying the magnetic force conditions inside the chip, which were obtained by adjusting the magnet distance from the wall.

Beads were introduced at t = 0 s into the domain at a constant flow rate of 500 s^−1^ for scenarios 1 and 2, which corresponds to a concentration of approximately 0.3 g·L^−1^, a concentration range commonly found in most of bead bioapplications^10^. For these scenarios, the beads were randomly injected through the diameter of the blood inlet, as presented in Fig. 2. For scenario 3, the increased simulation runtimes that are required to track the effects of the bead volume limited the number of beads considered to 3. These three beads were injected into the microchannel through the same inlet (Fig. 2) after the fluid flow reached the steady state condition. For all cases, the beads were introduced into the fluid domain with an initial velocity equal to the blood solution. The total simulation time was kept at 0.5 s for all simulations. The commercial solver employed for the three scenarios adjusts the time step to be as large as possible during runtime. For scenarios 1 and 2, the position of the beads was tracked at every 0.0005 s and the time step for these simulations was around 10^−5^ s. For scenario 3, the time step ranged from 10^−8^ and 10^−9^ s and the position of the beads was tracked at every time step.

The different models were solved using the commercial CFD software ***FLOW-3D*** (versions 11.1 and 11.2, Flow Science, Inc.). For scenarios 1 and 2, we used a Lagrangian particle model and a specific mass flowrate of beads was introduced into the upper inlet. However, for scenario 3, explicit modeling of moving objects was used and only the motion of three beads was modelled. The magnetic force, including the field and gradient equations, were solved in an external FORTRAN subroutine compiled in ***Visual Studio*** 2013 (Microsoft). This subroutine was linked to the ***FLOW-3D*** hydrodynamics solver while running and sent the components of the **F**_**m**_ to the solver at every time step. The simulations were performed on a 48-core workstation with 128 GB of RAM.

## Results and Discussion

### Bead separation results: One-way coupling versus two-way coupling

**Figure 3:**
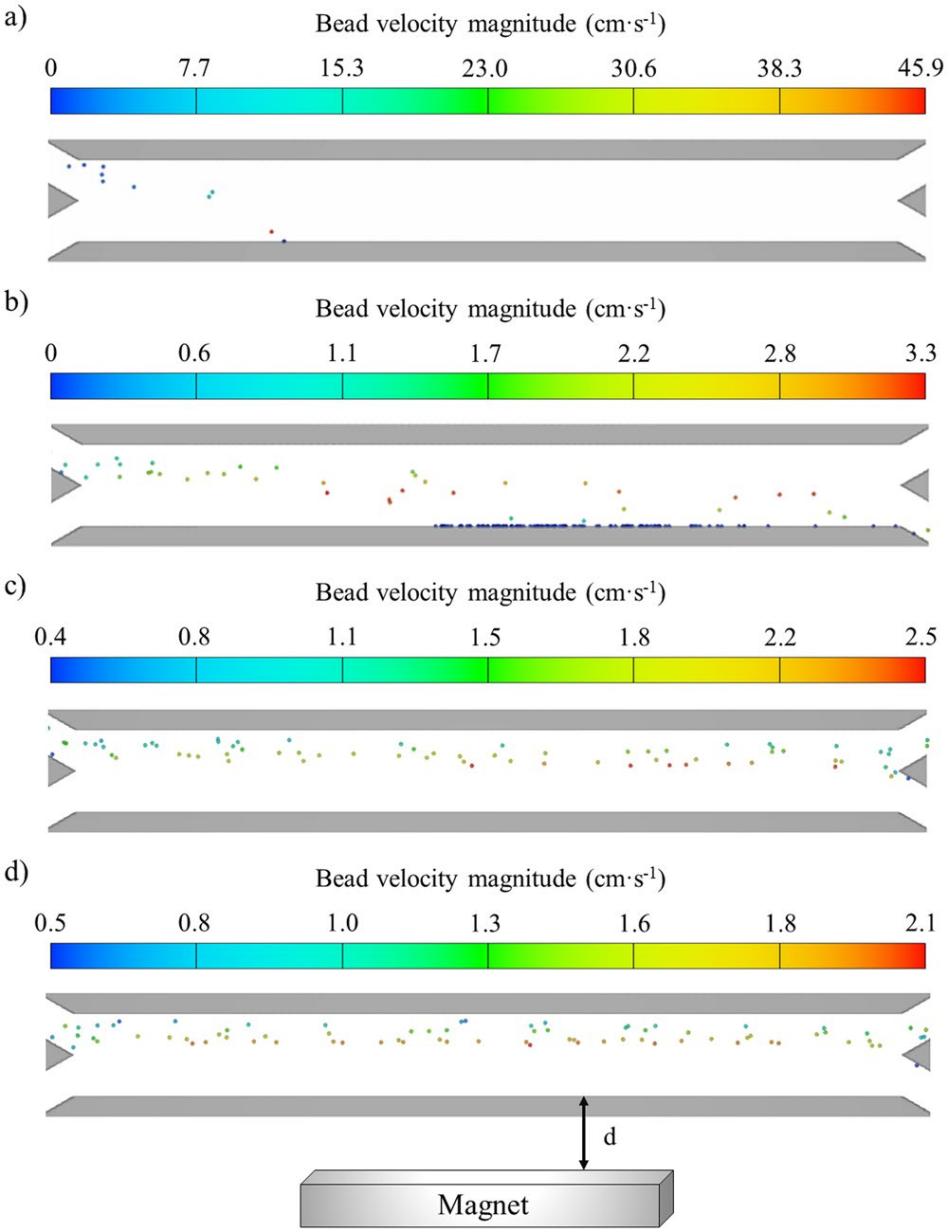
Bead trajectories for different magnetic field conditions, magnet placed at different distances “d” from the channel: (**a**) d = 0; (**b**) d = 1 mm; (**c**) d = 1.5 mm; (**d**) d = 2 mm.

In this section, the trajectories of the beads under different magnetic field conditions are analyzed and the average magnetic force required for complete separation is estimated. The trajectories of the beads, as they move from the biological fluid to the buffer solution, as a function of the distance of the magnet to the channel wall are shown in Fig. 3. In this case, only the results for scenario 1 (one-way coupling) are presented since the difference in bead locations inside the microchannel at every time step are not severely modified after including the two-way coupling (scenario 2). It should be noted that, although for scenario 3 we could only model the trajectories of three beads, the results regarding the bead trajectories as a function of the magnet distance “d” are consistent with the ones shown in Fig. 3, as reported in our previous work^18^.

As depicted in Fig. 3, when the distance “d” between the magnet and the channel is 1 mm or less, all the beads are deflected from blood to the buffer stream. However, larger distances resulted in the incomplete separation, which is in agreement with our previous calculations^18^. It should be noted that the magnetic field is very high when the magnet is place next to the wall (d = 0), reaching fields of around 450 mT. However, the field decays rapidly with d, in a nonlinear fashion, being practically negligible for d ≥ 2 mm. This field variation with d causes a decrease of the average magnetic force experienced by the beads, which varies from 13 nN to 0.022 nN as the magnet distance increases from 0 to 2 mm. Since the fluidic forces are held constant for all the simulations (the drag force is approximately 1.65 nN), as the magnetic force decreases, the beads remain in the upper branch of the channel and exit the device through the blood outlet.

As it is shown in Fig. 3(a), the magnetic force exerted on the beads is very strong for a magnet distance equal to 0, with a maximum value of 13 nN. For that condition, all the beads are permanently trapped at the same point (x = −500 µm, z = −100 µm). This point coincides with the highest gradient region, located at the x-coordinate at the magnet’s left edge. Furthermore, under this high magnetic force, the bead velocity increases dramatically as the distance from the wall decreases, reaching the lower wall of the channel at velocities on the order of 40 times higher than their initial velocity. Nevertheless, after reaching the wall, the beads are trapped at their impact point with zero velocity. This is due to the negligible drag force exerted on them in the proximity of the channel wall (i.e. almost zero fluid velocity because of the laminar flow regime) and the high magnetic force experienced by the beads at that point.

Upon moving the magnet 1 mm away from the channel wall (Fig. 3(b)), the magnetic gradient is more homogeneously distributed inside the microdevice above the surface of the magnet pole (with average magnetic forces of approximately 0.5 nN). Thus, the beads are deflected along the channel length rather than trapped at one location. Under these magnetic conditions, the particles are still allowed to move towards the outlet after reaching the wall, although their velocity is low. However, when the magnet distance d is greater than 1 mm (Fig. 3(c,d)), the force generated by the magnet drastically diminishes, with average magnetic forces lower than 0.1 nN. In this case, bead recoveries between 10% and 40% are obtained for d = 1.5 mm and d = 2 mm, respectively. Moreover, as the distance d increases, bead velocities decrease and take values very similar to the fluid velocity, as depicted in Fig. 3.

**Figure 4:**
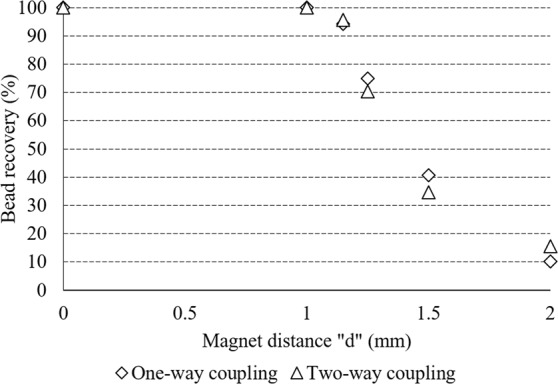
Separation efficacy as a function of the magnet distance. Comparison between one-way and two-way coupling.

On the other hand, in Fig. 4 we show the percentage of beads recovered at the lower outlet of our device for scenarios 1 and 2. It should be noted that when comparing the recovery efficacies obtained with one-way and two-way coupling, the results are very similar. For example, for magnet distances equal to 2 mm, recoveries of 10.2% and 15.5% are obtained for scenarios 1 and 2, respectively. However, higher recovery values are obtained for the one-way coupling scenario at distances d equal to 1.25 and 1.5 mm. Overall, the difference in bead recovery between scenarios 1 and 2 is lower than 6% for all the magnet locations calculated in this work. Therefore, we can assume that the small difference in the particle recovery obtained with both models is due to the randomized initial bead distribution at the entrance of the channel, which is different for every simulation, rather than a difference in the forces acting on the beads. Due to the high number of beads tracked for these simulations (more than 200), the initial distribution only causes a small variation of the results.

The similar results for one-way and two-way coupling are also due to the fact that the two-way coupling introduces the bead velocities into the Navier Stokes momentum equation, with relatively high magnitudes at high magnetic forces (as seen in Fig. 3, bead acceleration is the highest for d = 0). For the two-way coupling under high magnetic forces, the fluid flow is significantly perturbated, which renders the fluidic resistance acting on the beads lower. However, since all the beads are easily deflected at high magnetic forces, the lower fluidic force does not have an impact on the recovery rates in comparison to the one-way coupling. Furthermore, we believe that there is no difference in the forces acting on the beads for both scenarios. This is because if there was a difference in the force balance, the recovery obtained with the two-way coupling would be higher than the value obtained with the one-way coupling due to the flow perturbation and this is not observed for all the conditions tested but only for a few. Nonetheless, the high flow field perturbation does have an impact on the flow patterns of the phases, especially when the beads are crossing the interface, which is studied in the following subsection.

### Bead-fluid interactions: Two-way coupling versus two-way coupling including bead volume effects

In this section the modification of the flow patterns during the magnetophoresis are presented for the scenarios 2 and 3, in order to identify the effect of bead-fluid interactions on the separator performance. Scenario 1 (one-way coupling) is taken as a base case for comparison.

**Figure 5:**
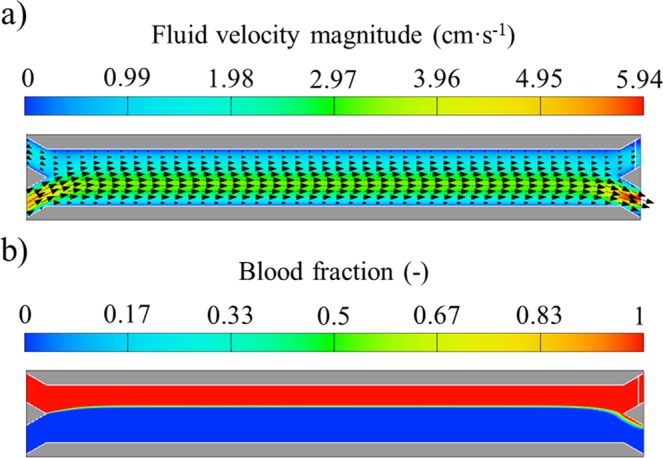
(**a**) Fluid velocity magnitude including velocity vectors and (**b**) blood volumetric fraction contours with magnet distance d = 0 mm for scenario 1 (t = 0.25 s).

In Fig. 5 the velocity field and the flow patterns for scenario 1 are shown for a magnet distance of d = 0. This distance was chosen because of the high bead acceleration that it causes. Nevertheless, this effect does not have an impact on the flow patterns as seen from the figures. The flow field after the development of the laminar velocity profile (t = 0.25 s) is shown in Fig. 5(a,b). It should be noted that the time necessary to achieve the steady state condition is less than 0.15 s. After that time, the fluid velocity field is not modified during the magnetophoresis process because the beads do not interact with the fluid. Therefore, for all the magnet positions tested in this work (not shown in the figure), the fluid flow remains unchanged after reaching the steady state. For this scenario, the velocity vectors are parallel to the x-axis, and the bead motion does not modify them, resulting in the desirable outcome of a stable interface between the fluids. It should be noted that the water phase flows at a higher velocity than blood, which causes the velocity flow field depicted in Fig. 5(a).

**Figure 6:**
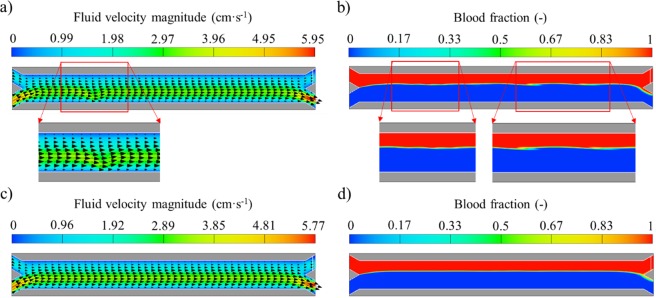
Fluid velocity magnitude including velocity vectors and blood volumetric fraction contours for scenario 2: (**a**,**b**) Magnet distance d = 0 mm at t = 0.4 s; (**c**,**d**) Magnet distance d = 1 mm at t = 0.4 s.

On the other hand, the fluid velocity vectors and the effect of bead magnetophoresis on the flow patterns for the two-way coupling model are shown in Fig. 6 for both relatively high (Fig. 6(a,b)) and low (Fig. 6(c,d)) magnetic fields and forces. In Fig. 6(a,b)), the effect of bead separation on the flow field is shown for high magnetic force fields (corresponding to a magnet distance d = 0 mm). In this case, the direction of the velocity vectors is slightly modified at the region where the magnetic gradient is the highest (x = −500 µm, z = −100 µm), which is magnified in the figure. Nonetheless, the velocity magnitude does not change in comparison to the one-way coupling model, because for this scenario the bead-fluid coupling is not as accurate as in the third scenario. However, the flow patterns are modified and the interface between the two fluids is perturbated for this condition, as shown in Fig. 6(b). As mentioned before, this is due to high bead accelerations that are added to the Navier Stokes fluid momentum equation. By comparing scenarios 1 and 2, and by comparing Fig. 6(a–d), it can be observed that this change of the momentum equation appears to affect more the volumetric fraction of the fluid phases rather than the velocity field for this model. Finally, it should be noted that the interface perturbations persisted for subsequent times under this condition.

However, when the beads undergo only modest acceleration (which happens when the magnetic force is reduced as the magnet distance increases, as seen in Fig. 6(c,d) for d = 1 mm), the momentum of the fluid is not significantly affected. This explains the absence of perturbation of the velocity vectors as seen in Fig. 6(c). In this case, both the velocity magnitude and flow field are substantially the same as observed for the one-way coupling model. However, for this magnet distance, the flow patterns are slightly modified. Although the interface remains clear and no waves are observed, the phase separation at the channel outlet improves in comparison with the previous condition. Nonetheless, they are very similar to the ones observed in Fig. 5 for our benchmark case, i.e. one-way coupling.

Finally, it should be noted that the rest of the magnetic conditions solved with the two-way coupling model are not shown in Fig. 6 because when d > 1 mm, very similar flow patterns are observed. Nonetheless, bead separation with the magnet at these distances is incomplete, as presented in the previous subsection.

**Figure 7:**
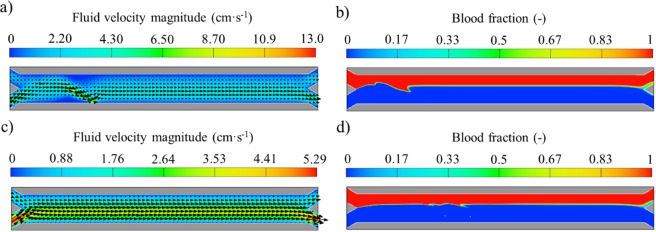
Fluid velocity magnitude including velocity vectors and blood volumetric fraction contours for scenario 3: (**a**,**b**) Magnet distance d = 0; (**c**,**d**) Magnet distance d = 1 mm.

Furthermore, when the volume of the beads is taken into account in the flow field equations (scenario 3), the liquid displacement due to each bead as it moves from the blood stream to the buffer solution has a significant effect on both the vectors and the volumetric fraction of the fluid phases, especially under high magnetic forces. The flow field results for scenario 3 are shown in Fig. 7. For high magnetic force fields, most of the flow velocity vectors are highly perturbated and change around the beads as seen in Fig. 7(a). On the other hand, for the two-way coupling model (scenario 2) only the flow vectors close to the lower wall are slightly changed for this magnetic field condition. Moreover, the velocity magnitude increases up to 13 cm·s^−1^ for this scenario (remaining below 6 cm·s^−1^ for the two-way coupling model as seen in Fig. 6(a)). The difference in the velocity magnitude for scenarios 2 and 3 is mostly attributed to the finer mesh necessary for resolving the beads in scenario 3. More specifically, for accounting for the bead volume effects in scenario 3, the bead diameter should cross multiple cells. However, in scenarios 1 and 2, the size of the mesh cells is smaller than the beads (which are modeled as points). Therefore, for scenario 2, the coarser mesh does not allow for such velocity extrema since the 2-way coupling is acting on a larger volume of fluid.

As also seen in Fig. 7(b), for this magnetic field conditions, the interface between fluids is highly altered for this scenario. This is caused by the inclusion of the volume of the beads into the governing equations. Specifically, in scenario 3 both the momentum equation and the continuity equation are modified. Phase separation at the Y junction outlet is also affected as the buffer solution enters the upper outlet, which may be due to the displacement of buffer by the volume of the separated beads. This phenomenon was not observed for scenarios 1 and 2. However, recent studies have reported the change in the interface location as magnetic materials are accumulated at the wall, which hinders the co-flow with time^42^. It should be noted that the time required for separating the three beads is less than 20 milliseconds for this magnetic field condition. Nonetheless, the effect of bead magnetophoresis on the fluid velocity vectors can be neglected under relatively low magnetic fields (d ≥ 1 mm) as seen in Fig. 7(c). This is due to the slow bead deflection when the magnetic and fluidic forces are of comparable magnitude. Therefore, under these circumstances, bead acceleration is negligible and bead velocity remains constant for all simulation times. In this case, the time required for the deflection increases to almost 100 milliseconds. This is translated into the flow patterns presented in Fig. 7(d). The interface at the time when the beads are crossing it is not affected under these magnetic conditions, which favors the fluid co-flow.

**Figure 8:**
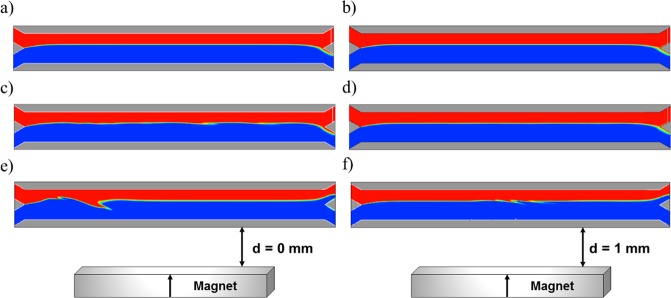
Blood volumetric fraction contours. Scenario 1: (**a**) Magnet distance d = 0 and (**b**) Magnet distance d = 1 mm; Scenario 2: (**c**) Magnet distance d = 0 and (**d**) Magnet distance d = 1 mm; and Scenario 3: (**e**) Magnet distance d = 0 and (**f**) Magnet distance d = 1 mm.

In summary, the flow field and phase separation are similar for the three scenarios under low magnetic field conditions. However, the flow field is altered when high magnetic forces are employed which in turn affects the phase separation and fluid co-flow. This effect on the flow patterns is presented in Fig. 8 where the phase separation under the magnetic field conditions that achieve 100% of bead separation (d = 0 and d = 1 mm) is presented for the three scenarios after the steady state condition is stablished. Furthermore, the mixing of the phases was quantified at each outlet for the different scenarios. As shown in Fig. 8(a,b), the one-way coupling model does not predict any change in the flow patterns for the magnet distances tested in this work. However, as seen in Fig. 8(c), the two-way coupling produces a wavy interface along the channel length, however, this does not result in mixing at the outlets as reported from the blood fraction fluids. Therefore, blood loss or dilution at the outlets is negligible for this model for all the magnetic conditions simulated (Fig. 8(c,d)). On the contrary, when the beads volume is taken into account (scenario 3), water is introduced into the upper outlet and consequently blood is diluted up to 45%. This value changes slightly with the magnetic field conditions (as long as the separation of the magnetic beads is carried out, d ≤ 1.25 mm), which implies that the addition of material to the water phase displaces the interface between the two phases upwards (it creates different pressure drops at each branch of the channel), which, in turn, causes the presence of buffer solution at the upper outlet, as shown in Fig. 8(e,f).

## Conclusions

In this work, we have performed a flow-focused study of the magnetophoresis of micron-sized magnetic beads inside a continuous flow microfluidic device in order to provide a detailed analysis of the bead motion and its effect on the fluid flow. Bead magnetophoresis is modeled with a Lagrangian approach in a microseparator where the beads are continuously separated from blood and collected into an aqueous solution by the application of an external magnetic field provided by a rare-earth permanent magnet. The dominant magnetic and fluidic forces were included in the force balance to study bead motion and we quantified bead recovery as a function of the magnet distance to the channel wall. Different fluid perturbation scenarios were modelled: (i) one-way coupling between the beads and the fluid (i.e. bead motion does not affect the fluid flow), (ii) two-way coupling (i.e. full momentum transfer between the fluid and the beads, which were treated as point particles) and (iii) two-way coupling in which the volume of the beads was taken into account to include the corresponding fluid displacement.

Our results showed that bead recovery results for the one-way coupling model were not significantly modified after including the two-way coupling in the model. However, fluid velocity magnitude and vectors slightly varied after including the momentum transfer between the beads and the fluid only when the magnetophoresis is carried out under high magnetic force fields. Nonetheless, the flow field results for scenario 3 were significantly altered for high magnetic force fields, whereas the changes could be considered negligible for low magnetic field conditions.

Based on these results, we conclude that when relatively high magnetic force fields are applied for the recovery of magnetic beads, an accurate full flow-focused model should be solved since there is a risk of neglecting the effects that the motion and volume of the magnetic beads have on the flow field. However, this model is computationally expensive and takes substantially more time to run (in our case 2–3 weeks on a modern multicore workstation). When medium or low magnetic force fields are employed (in comparison to the average fluidic forces), the bead-fluid interactions could be omitted as they are negligible for these cases. Moreover, since the bead accelerations are not desirable for continuous-flow devices because of the flow alterations, the best alternative is to employ medium to low magnetic forces to carry out the magnetophoresis. And under these desirable circumstances, less accurate, experimentally validated^37^, and computationally inexpensive models could be employed (either scenarios 1 or 2, which took only a couple of hours to run on a modern multicore workstation).
